# Enhanced production and biodeinking application of β-glucosidase from *Aspergillus niger* S1 via submerged fermentation using office paper waste through conventional and statistical approaches

**DOI:** 10.1186/s12896-026-01139-9

**Published:** 2026-04-13

**Authors:** Doaa Ali, Mohamed S. Hasanin, Mohamed Abdelraof, Abdelmageed M. Othman, Ghada S. A. Abdel Karim, Alshaimaa M. Elsayed

**Affiliations:** 1 Agricultural Microbiology Dept., Agricultural and Biological Research Institute, National Research Centre, Cairo, 12622 Egypt; 2 Cellulose and Paper Department, National Research Centre, 33 El Bohouth St., Dokki, Giza, 12622 Egypt; 3 Microbial Chemistry Department, Biotechnology Research Institute, National Research Centre, Dokki, Giza 12622 Egypt; 4 Faculty of Biotechnology, German International University, Regional Ring Road, East Cairo, New Administrative Capital, Cairo, Egypt; 5 Molecular Biology Department, Biotechnology Research Institute, National Research Centre, Dokki, Giza, 12622 Egypt

**Keywords:** β-glucosidase, Production optimization, Statistical approaches, Office paper waste, Bio-deinking

## Abstract

**Background:**

β-glucosidases are highly versatile catalysts with significant potential in various industries, including wastepaper deinking, which is a sustainable alternative due to its high effectiveness and minimal environmental impact.

**Results:**

In this study, the optimal conditions for using submerged fermentation (SmF) to produce β-glucosidase from a newly isolated soil fungal species (*Aspergillus niger* S1) were improved. The main variables that influence the formation of β-glucosidase were examined, including temperature, pH, incubation period, nitrogen source, emulsifier concentration, and carbon source concentration. The production of β-glucosidase by the recently isolated *Aspergillus niger* S1 using office paper waste (OPW) as feedstock is encouraging because it reached its maximum production after just 3 days of incubation at pH 6.5 and 30 °C with office paper waste (15 g/L) serving as a carbon source at 1.5% (w/v), Tween 80 at 1.5% (v/v), and yeast extract as a nitrogen source. Using the CCD-based optimization approach, an enzyme yield of 3.35 U/mL β-galactosidase was achieved after 3 days of incubation at pH 6.0, with 1.5% (w/v) office paper waste and 1 g/L yeast extract. It was confirmed by the results that the β-glucosidase activity eliminated the ink particles and that this action improved the ash content, whiteness, and tensile strength.

**Conclusion:**

The findings showed that the investigated enzyme was a suitable green substitute for office paper waste deinking in the direction of sustainable waste management.

**Supplementary Information:**

The online version contains supplementary material available at 10.1186/s12896-026-01139-9.

## Introduction

Most solid waste management procedures include incineration and landfilling, which lead to the degradation of cellulosic wastes with the formation of dangerous substances such as greenhouse gases like carbon dioxide and methane [1]. These chemical compounds have a cascading impact on the atmosphere that could be linked to current changes in global climate patterns and loss of soil biodiversity [2–5]. A major component of municipal or agricultural solid waste is of plant origin. Annually, major volumes of cellulose waste materials are dumped, such as food, paper, garden, and plant waste. Lignocellulosic biomass feedstocks are attracting significant scientific interest for their potential in producing value-added products [6]. The use of lignocellulosic biomass feedstocks to manufacture value-added goods—such as fuels, fertilizers, food additives, pesticides, enzymes, aromatic compounds, pharmaceuticals, detergents, organic acids, and plastics—has attracted significant attention [7]. The choice of substrate for the synthesis of enzymes is mostly determined by its accessibility and viability from an economic standpoint [8]. Cellulose is the most abundant polymer in the biosphere. Cellulases, which are responsible for cellulose hydrolysis, are made up of a diverse variety of enzymes with varied specificities for hydrolyzing glycosidic linkages. β-glucosidases are one of the cellulase enzymes that hydrolyze glucose dimers from previous enzymatic actions into glucose.

Cellulases are a group of hydrolytic enzymes responsible for breaking down waste cellulose, the most abundant polymer in the biosphere. Depolymerization of cellulose to glucose requires the synergistic action of three enzymes due to its highly ordered and crystalline structure [9–11]. The three main groups of cellulases—endoglucanases, exoglucanases, and β-glucosidases—hydrolyze the β-1,4-glycosidic bonds in cellulose, producing glucose, cellobiose, and cello-oligosaccharides as the main products [12]. Endoglucanases cleave within the cellulose chain, releasing glucose, cellobiose, and cello-oligosaccharides. Exoglucanases processively attack the chain ends, predominantly releasing cellobiose. In the final stage, β-glucosidases complete the hydrolysis process by converting cellobiose and cello-oligosaccharides into glucose [13]. β-glucosidases are highly versatile catalysts with significant potential in various industries, including wastepaper deinking [14], antimicrobial agents [15], pharmaceuticals, cosmetics, detergents, and food industries [16, 17].

Mixed office paper waste generated from photocopiers, printouts, and other office sources is a valuable source of premium paper fibers. However, its recycling is challenging because it contains diverse inks that act as a barrier during hydrolysis, preventing cellulase enzymes from accessing the paper material [18]. Traditional chemical deinking techniques address this issue but pose numerous disadvantages, including the use of toxic chemicals, high costs, and the generation of ecologically harmful byproducts. These methods often employ sodium hydroxide for pH adjustment, surfactants for ink dispersion, and strong oxidative agents to decompose ink [19].

As a sustainable alternative, enzymatic deinking has gained significant attention in the last decade due to its high effectiveness and minimal environmental impact [20, 21]. This approach improves deinking by partially hydrolyzing cellulose and freeing ink particles from the pulp fiber surface, potentially eliminating the need for intensive processes like dewatering and re-floatation [22]. For this purpose, various enzymes including cellulases, hemicellulases, laccase, and pectinase are used alone or in combination [23]. The commercial feasibility and eco-friendliness of enzymatic deinking are drawing industrial interest, with studies, such as one by Chandranupap and Chandranupap [24], demonstrating its effectiveness in improving pulp brightness and significantly reducing residual ink concentration [25].

Papermaking is a complex process with different steps, from pulping to bleaching. All paper-making steps also use a huge amount of chemicals and water. Unfortunately, not all paper-making steps can be alternatively replaced with a biotechnological process [14]. However, the bleaching process could be carried out using some enzymes in the crude form, such as cellulases, lignin peroxidases, and hemicellulose [26]. On the other hand, the reuse of printed paper is a big sector now that has developed to avoid the cutting of trees to start paper manufacturing from zero point. Indeed, the biotechnology sector is entering this field with force, with some challenges that scientists must now overcome using different forms of enzymes, such as pure, crude, immobilized, and so on, to release the residual ink from the paper matrix, and this process is called bio-deinking. In this context, β-glucosidase is a key enzyme in the process of bio-deinking, which acts on the β-1,4 linkages between glucosyl residues. They catalyze the hydrolysis of the corresponding glycoside’s terminal, nonreducing β-linked monosaccharide residues. β-glucosidase contributes to the comprehensive degradation of cellulose, which is essential for loosening ink particles bound to the fibers. This action enhances the efficiency of ink removal during the flotation stage of paper recycling [27].

Species of *Aspergillus* and *Trichoderma* are well-known candidates for industrial applications due to their potential to produce a wide range of extracellular enzymes. However, for efficient cellulose degradation, a strain must possess highly active endoglucanases, exoglucanases, and β-glucosidases in ideal ratios [9, 28]. Significant progress in the enzymatic deinking of office paper waste and the optimization of enzyme production for pulp and paper applications has recently been demonstrated [29]. However, closing the gap between the production and use of enzymes continues to be a major challenge. These are frequently handled as distinct unit operations: application studies usually employ commercial enzyme preparations, while enzyme fermentation is optimized for maximum titer without taking downstream deinking performance into direct consideration. This separation ignores possible synergies and might not result in a process that is comprehensively optimized for industrial viability, where elements like overall process time, final pulp quality (such as whiteness and strength), and scalability are crucial. The current investigation aims to optimize the various parameters affecting the biosynthesis of *Aspergillus niger* S1 β-glucosidase under submerged fermentation using office paper waste and assessing its eco-sustainable deinking efficiency.

## Materials and methods

### Chemicals and materials

Dinitrosalicylic acid (DNS), 4-nitrophenyl-β-D-glucopyranoside (*p-*NPG), Czapek-Dox, and Potato dextrose broth and agar media were provided by Sigma-Aldrich (USA). Other chemicals used throughout the current study were of analytical grade. Mixed OPW was gathered from the offices of the National Research Centre. Laser jet printers and laser toner were utilized for the ink. OPW was mechanically disintegrated into small fragments and incorporated into the fermentation medium as a carbon source for enzyme production by the fungal strain.

### Isolation and molecular identification of the selected fungal strain

A serial dilution technique was used for the isolation of different fungal isolates from different planted muddy soil samples collected from an agricultural field located at 30° 32′ 49.06″ N, 31° 16′ 2.4″ E; Kafr Shukr city, Qalyubia Governorate in the Nile Delta region of Egypt. The soil was characterized as loamy with a neutral pH and moderate organic matter content. Fungal isolation from soil samples was performed using the standard plate dilution method. Approximately 10 g of soil was suspended in 90 mL of sterile distilled water and serially diluted. Aliquots (100 µL) from appropriate dilutions were spread onto Potato Dextrose Agar (PDA) plates supplemented with ampicillin (100 µg/mL) to inhibit bacterial growth and favor fungal development. The plates were incubated at 28 ± 2 °C for 3–7 days. Emerging fungal colonies with distinct morphological features were subcultured repeatedly on fresh PDA plates to obtain pure isolates. The different isolates were then grown on modified Czapek-Dox liquid medium containing printed office paper waste (2%) as a carbon source for fungal growth, with the composition of (g/L): printed office paper waste, 20.0; NH_4_Cl, 1.26; KH_2_PO_4_, 1.0; MgSO_4_.7H_2_O, 0.5; KCl, 0.5 and Tween-80, 2–8% (v/v) at pH 5.0. The secreted β-glucosidase enzyme was quantified according to the standard reaction conditions (mentioned in the enzyme assay section), and the highest enzyme producer was selected for further screening and identification.

Morphological characteristics were examined using a scanning electron microscope (JSM 5600 LV; JEOL, Tokyo, Japan) and confirmed by molecular identification. The selected high-enzyme-producing fungal isolate was grown in Potato Dextrose broth for 3 days. Then, following the manufacturer’s instructions, the genomic DNA was isolated using a Gene JET Genomic DNA purification kit (Thermo Scientific, Lithuania). Internal Transcribed Spacer 1, Forward (ITS1F) primer (5′-CTTGGTCATTTAGAGGAAGTAA-3′), and reverse primer ITS4R (5′-TCCTCCGCTTATTGATATGC-3′) were used to amplify the ITS gene. The Macro Gene company in South Korea purified and sequenced the amplified PCR product. The Finch TV 1.4.0 program was used to modify the raw sequencing data (contig and peak chromatogram verification). The National Center of Biotechnology Information (NCBI)’s Standard Nucleotide Basic Local Alignment Search Tool (BLASTN) (Rockville Pike, Bethesda, MD, USA) was used to analyze the strain’s ITS sequences. The ClustalW 2.1 tool was used to align multiple sequences. Molecular Evolutionary Genetics Analysis Version 11 (MEGA-11) used the neighbor-joining technique to build the phylogenetic tree [30].

### Cultivation of organisms and maintenance medium for stock fungal culture

The following composition (g/L) of modified solid Czapek-Dox medium was used to develop and maintain the fungal isolate: glucose, 20.0; NaNO_3_, 2.0; KH_2_PO_4_, 1.0; MgSO_4_.7H_2_O, 0.5; KCl, 0.5; and Agar, 20.0 [31]. The medium’s pH was adjusted to pH 5.0 and sterilized by autoclaving at 121 °C for 20 min at 15 psi (≈ 1.5 atm).

### Crude β-glucosidase production

The identified isolate (*Aspergillus niger* S1) was grown on modified Czapek-Dox liquid medium containing printed office paper waste (2% w/v) as a carbon source for fungal growth, with the composition of (g/L): printed office paper waste (~ 5 × 5 mm), 20.0; NH_4_Cl, 1.26; KH_2_PO_4_, 1.0; MgSO_4_.7H_2_O, 0.5; KCl, 0.5 and Tween-80, 2–8% (v/v) at pH 5.0, and sterilized by autoclaving at 1.5 atmosphere and 121 °C for 20 min. Under aseptic circumstances, three PDA discs (6 mm) were used to inoculate 250 mL Erlenmeyer flasks with 50 mL of sterile medium each. The flasks were incubated at 30 °C for three days on a shaker (New Brunswick Scientific Co. Inc., Edison, N. J. USA) at 150 rpm. The fungal mycelia were then filtered using filter papers (Whatman No. 1), and the cell-free filtrate (CFF) was used as the source of enzyme.

### Enzyme assay

For the determination of β-glucosidase activity, the assay was conducted by mixing 500 µl of 5.0 mM *p-*NPG prepared in sodium citrate buffer (50 mM, pH 4.8) and 400 µl of enzyme solution. The reaction was stopped by adding 500 µl of 20% sodium carbonate after 30 min of incubation at 45 °C. The absorbance of the reaction mixture was measured at 405 nm, following the method of Ng et al. [32] with appropriate modifications. The standard calibration curve with known concentrations of p-nitrophenol was prepared. The enzyme stock was diluted appropriately to ensure activity measurements fell within the linear range of the assay (0–300 µM p-nitrophenol, R² > 0.99). Blanks without substrate and blanks without enzyme were included to correct for non-enzymatic hydrolysis and background absorbance, respectively. One unit of β-glucosidase activity is expressed as the amount of enzyme that releases 1 µmol of *p*-nitrophenol per minute under assay conditions.

Endoglucanase activity was measured in a reaction mixture (1 mL) containing 0.5 mL of enzyme and 0.5 mL of 1% (w/v) CMC prepared in citrate buffer (50 mM, pH 4.8). The reaction mixture was incubated at 50 °C for 10 min [9]. Exoglucanase (FPase) activity was measured following the method described by Chandra and Reddy [33]. The reaction mixture (1 mL) consisted of 0.5 mL of enzyme, 0.5 mL of citrate buffer (50 mM, pH 4.8), and 50 mg of Whatman No. 1 filter paper strips as substrate. The mixture was incubated at 50 °C for 60 min. The Dinitrosalicylic acid (DNS) method [34] was used to estimate the enzymatic hydrolysis products of CMC and filter paper by measuring the absorbance of the released glucose at 575 nm with glucose as the standard. One unit (U) of enzyme activity was defined as the amount of enzyme required to release 1 µmol of glucose per minute under the assay conditions. Protein concentration was determined by the Bradford method using bovine serum albumin (BSA) as the standard protein [35].

### Optimization of β-glucosidase production

Physiological and culture parameters were optimized using a one-factor-at-a-time (OFAT) strategy to maximize the β-glucosidase enzyme’s output. The impact of several fermentation parameters, including temperature, incubation duration, pH level, carbon concentrations, emulsifier concentration, and nitrogen sources, was examined.

The optimization of nutritional and environmental factors is crucial for maximizing microbial biomass and subsequent enzyme yield [36]. Unlike the traditional OFAT method, which is not only time-consuming and costly but also fails to reveal interactions between variables [5, 37], Response Surface Methodology (RSM) can effectively model these complex relationships. In this study, RSM was employed to optimize β-glucosidase production by *Aspergillus niger* S1. Initial OFAT studies were first conducted to determine the experimental ranges for the key independent variables. Subsequently, a Central Composite Design (CCD) was applied to model the process. Four critical parameters were selected for optimization: incubation time (A), pH (B), office paper waste concentration (C), and yeast extract concentration (D). The CCD consisted of 30 experimental runs, including six center points, with each factor evaluated across five levels (-2, -1, 0, + 1, +2). The resulting β-glucosidase activity (Y) was treated as the dependent response variable. The data were analyzed using Design Expert^®^ software (Version 7.0.0) to fit a regression model and investigate the interactions between the parameters, as detailed in Table 1. The general form of the quadratic model used for the Response Surface Methodology (RSM) is:

**Table 1:** Optimization of β-glucosidase production using central composite design

Run	Incubation time (days)	pH value	Office paper waste (g/L)	Yeast extract (g/L)	ß-glucosidase activity (U/mL)	
					Actual value	Predicted value
1	3 {0}	6.5 {0}	1.0 {0}	1.5 {0}	2.698	2.544
2	4 {1}	7.0 {1}	1.5 {1}	1 {-1}	2.435	2.220
3	4 {1}	7.0 {1}	0.5 {-1}	1 {-1}	1.914	2.000
4	3 {0}	6.5 {0}	1.0 {0}	1.5 {0}	1.929	2.544
5	4 {1}	6.0 {-1}	0.5 {-1}	2.0 {1}	2.019	1.702
6	4 {1}	6.0 {-1}	0.5 {-1}	1.0 {-1}	2.091	2.419
7	2 {-1}	6.0 {-1}	0.5 {-1}	2.0 {1}	1.764	2.074
8	4 {1}	6.0 {-1}	1.5 {1}	2.0 {1}	1.696	1.792
9	3 {0}	6.5 {0}	1.0 {0}	1.5 {0}	2.537	2.544
10	3 {0}	6.5 {0}	2.0 {2}	1.5 {0}	2.545	2.297
11	2 {-1}	6.0 {-1}	0.5 {-1}	1.0 {-1}	2.721	2.521
12	4 {1}	6.0 {-1}	1.5 {1}	1.0 {-1}	2.541	3.042
13	3 {0}	6.5 {0}	0.0 {-2}	1.5 {0}	2.157	2.090
14	3 {0}	7.5 {2}	1.0 {0}	1.5 {0}	2.950	2.689
15	5 {2}	6.5 {0}	1.0 {0}	1.5 {0}	1.969	1.415
16	4 {1}	7.0 {1}	1.5 {1}	2.0 {1}	1.354	1.775
17	3 {0}	5.5 {-2}	1.0 {0}	1.5 {0}	2.930	2.876
18	3 {0}	6.5 {0}	1.0 {0}	1.5 {0}	2.546	2.544
19	2 {-1}	7.0 {1}	1.5 {1}	1.0 {-1}	1.931	2.468
20	3 {0}	6.5 {0}	1.0 {0}	1.5 {0}	2.992	2.544
21	2 {-1}	7.0 {1}	0.5 {-1}	1.0 {-1}	2.353	2.351
22	2 {-1}	6.0 {-1}	1.5 {1}	2.0 {1}	1.926	2.061
23	4 {1}	7.0 {1}	0.5 {-1}	2.0 {1}	1.564	2.088
24	3 {0}	6.5 {0}	1.0 {0}	2.5 {2}	2.162	1.992
25	2 {-1}	6.0 {-1}	1.5 {1}	1.0 {-1}	3.470	3.041
26	3 {0}	6.5 {0}	1.0 {0}	0.5 {-2}	3.029	2.883
27	2 {-1}	7.0 {1}	0.5 {-1}	2.0 {1}	2.991	2.710
28	3 {0}	6.5 {0}	1.0 {0}	1.5 {0}	2.563	2.544
29	2 {-1}	7.0 {1}	1.5 {1}	2.0 {1}	2.527	2.294
30	1 {-2}	6.5 {0}	1.0 {0}	1.5 {0}	1.798	2.036

Five levels (− 2, − 1, 0, 1, 2) of each studied parameter in the CCD were used to examine the statistical potential of the enzyme production process. Thirty runs with six center points were conducted to look into the interactions between the parameters under investigation

$$Y{\mkern 1mu} = {\beta _0} + \Sigma {\beta _i}{X_i} + \Sigma {\beta _i}_i{X_i}^2 + \Sigma {\beta _i}_j{X_i}{X_j}$$

Where Y is the predicted response, β₀ is the constant coefficient, β_i_ are the linear coefficients, β_i__i_ are the quadratic coefficients, β_i_ⱼ are the interaction coefficients, X_i_ and Xⱼ are the independent variables.

### Bio-deinking

Pretreatment of the mixed OPW was carried out as follows: OPW (a piece measuring about 2 × 2 cm) was washed 3 times with distilled water and suspended in a concentration of 5 g of OPW in 100 mL of distilled water. After 1 h, the fibers were mixed with a hand blender for 15 min to form a homogeneous fiber suspension, then filtered and dried at 70 °C overnight. The above-mentioned OPW was autoclaved before the bio-deinking process. The enzymatic treatment was carried out using 15 mL of the cell-free filtrate (CFF) at an enzyme concentration of 2.98 U/mL, added to 5 g of autoclaved OPW, which was then incubated for 3 days at 28 ± 2 °C and 150 rpm. To remove any medium components, the enzymatically treated OPW was filtered through Whatman No. 1 filter paper and then rinsed three times with distilled water. Following filtering, the enzymatically treated OPW was dried overnight at 70 °C in an oven. The control samples followed the procedures described above, with the CFF replaced in the sodium citrate buffer.

#### Paper sheets preparation

A standard Valley-type laboratory beater (Voith Inc., Appleton, WI, USA) was used to beat the pulps at a consistency of 2% (w/w) [38]. Untreated and enzymatically treated OPW were separately suspended in water at 2% (w/w) pulp consistency and mechanically agitated for 5 min at 1000 rpm. Paper sheets were prepared using a laboratory Rapid-Kothen sheet former (ISO 5269) and dried to obtain sheets weighing approximately 2 g each [39]. The sheets were pressed for five minutes at 420 kPa and 80 °C following the forming step. Before characterization, the hand sheets were conditioned for one day at 25 °C and 50% relative humidity, and the results are recorded as the mean of three readings with standard deviation.

#### Hand sheet characterization

##### Mechanical properties

At a constant crosshead speed of 2.5 cm/min, a universal testing machine (LR10K; Lloyd Instruments, Fareham, UK) equipped with a 100-N load cell was used to evaluate mechanical parameters (tensile strain) following the TAPPI T494-06 standard procedure. In the test, strips with a 20 cm length, 15 mm width, and 10 cm spread were utilized [22].

##### Optical (Brightness) properties

Using the Spectro Color LMG 183 colorimeter (Dr. Bruno Lange GmbH & Co. KG), the optical characteristics of the produced paper samples were investigated. Whiteness was evaluated following TAPPI standard method T452. Deinking ability was determined using the following formula:

$${\rm{Deinking}}\;{\rm{ability}} = {{({B_{{\rm{treated}}\;{\rm{sample}}}} - {B_{{\rm{untreated}}\;{\rm{sample}}}})} \over {{B_{{\rm{untreated}}\;{\rm{sample}}}}}} \times 100$$

where the whiteness of the samples is represented by B.

##### Paper sheet ash content

The ash content of the samples was assessed gravimetrically using the thermal method treatment at 800 °C in a muffle furnace for 2 h. The quantity of ink in the acquired sheets and the amount of ash in the office paper waste was determined using TAPPI procedures T 211.

### Statistical analysis

Unless otherwise noted, every experimental work was conducted in triplicate, and the results were reported as the mean value ± standard deviation. Sigma Plot software (version 11.0) was used to create graphs. Statistical analyses of the bio-deinking analyses were carried out using Minitab^®^ 21.2, 2022. The data were shown as mean ± SE and calculated from at least three replicates (*n* = 3).

## Results and discussion

### Molecular identification of the selected fungal strain

In the current study, from fifty-two fungal isolates obtained from the mentioned soil samples, the isolate that presented the highest β-glucosidase activity was selected to do further identification procedures. The morphological characteristics of the isolate were identified using scanning electron microscopy (SEM). A thick mycelial network was formed by the hyphae, which seemed septate and extremely branching. Long and upright, the conidiophores ended in globose vesicles that emitted phialides. These phialides produced chains of spherical conidia with surfaces that ranged from smooth to slightly rough. These structural observations support the identification of the isolate as *Aspergillus niger*.

**Fig. 1:**
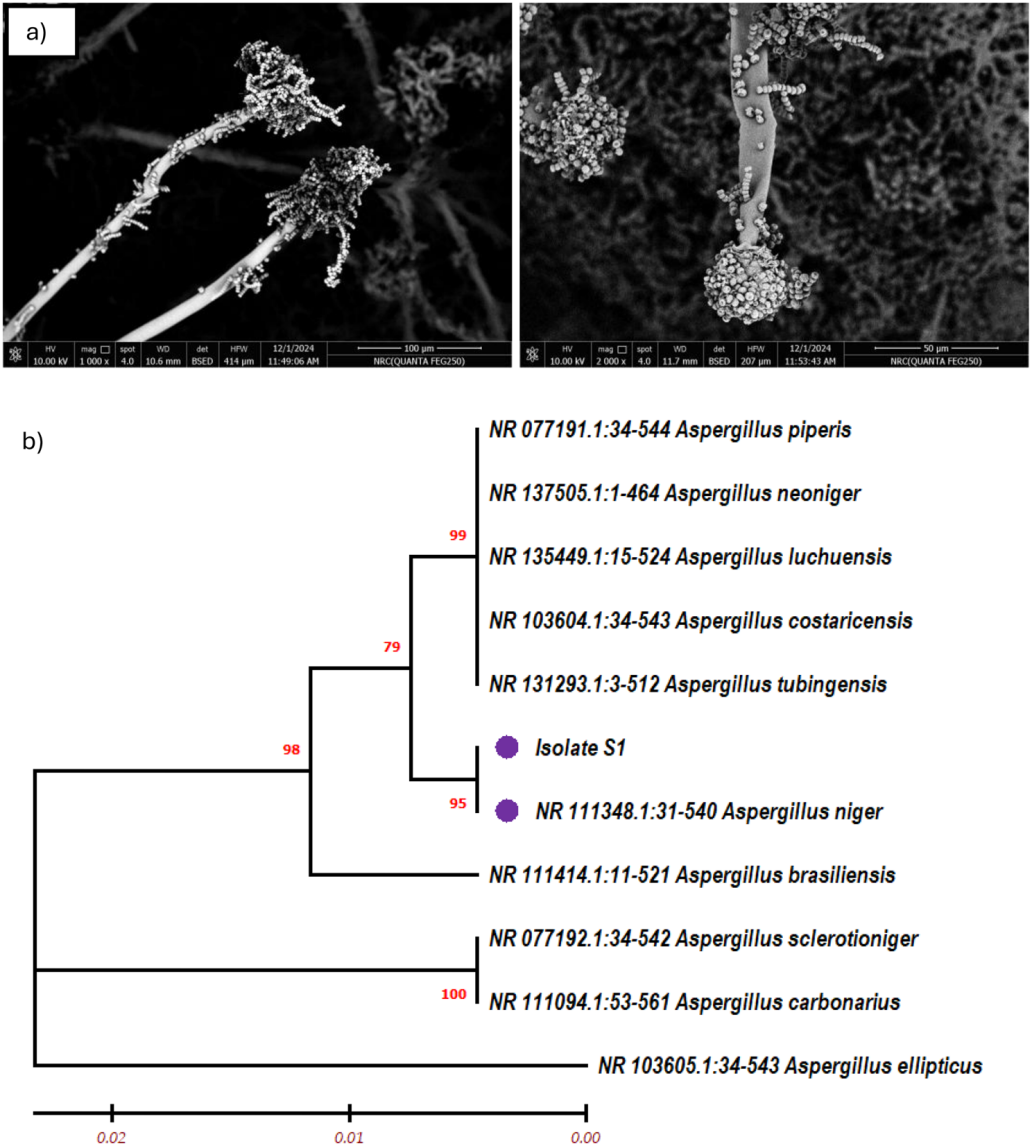
(**a**) Scanning electron micrographs (SEM) of the fungal isolate, (**b**) Phylogenetic tree based on partial ITS sequences, showing the relationship between isolate No (S1) *Aspergillus niger* and other species belonging to the genus *Aspergillus*. The tree was constructed using the MEGA-11 and neighbor-joining method

To confirm the identity of the fungal isolate, molecular identification was carried out by sequencing the ITS region of the ribosomal DNA. The ITS region of ribosomal DNA was amplified using the primers ITS1F and ITS4R to identify the most effective fungal isolate capable of producing β-glucosidase efficiently. The obtained ITS sequence was compared to those in the NCBI GenBank database using the BLASTN tool. The results revealed a 99.8% identity and high sequence similarity with several *Aspergillus niger* strains, indicating a close taxonomic relationship. The phylogenetic tree constructed using MEGA-11with the neighbor-joining method based on the ITS gene sequence of the isolate confirmed that the isolated fungus is *Aspergillus niger* S1 (Fig. 1a, b). With the accession number PV230693, the identified *Aspergillus niger* isolate S1 was registered in the GenBank (NCBI) database. Similarly, in another study, the strain *A. niger* JSC-093350089 was also identified through ITS region sequencing [40]. Hao et al. [41] revealed that the 18 S-rRNA encoding gene was used to identify *A. niger* AS3.4523, and that universal primers were used to amplify the gene using a genomic DNA template.

### Optimization of β-glucosidase production by *Aspergillus niger* S1

The optimization of β-glucosidase production was initially investigated using a one-factor-at-a-time (OFAT) approach. Various fermentation parameters were assessed individually to determine their influence on enzyme production. These included physical factors such as incubation time, pH, and temperature, as well as chemical factors like yeast extract (as a nitrogen source), Tween 80 (as a surfactant), and office paper waste concentration (as a carbon source). Then, based on the results of the OFAT approach, a response surface methodology design was applied to investigate the interaction between different effective parameters.

#### One-factor-at-a-time approach (OFAT)

##### Effect of incubation period on enzyme production

**Fig. 2:**
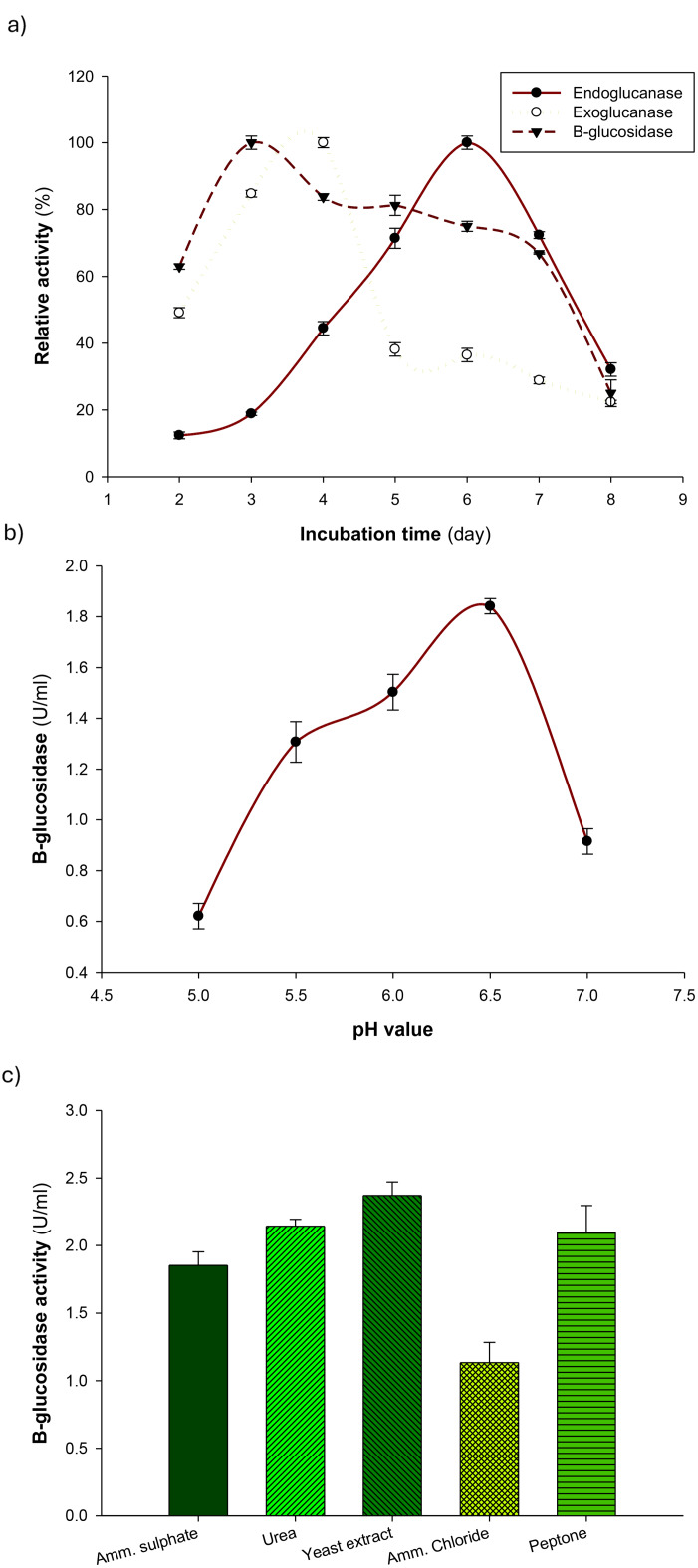
Effect of (**a**) incubation time, (**b**) pH, and (**c**) nitrogen sources on β-glucosidase production by the newly isolated *Aspergillus niger* S1. Error bars represent mean ± standard deviation

To optimize β-glucosidase production by *Aspergillus niger* S1, we first focused on identifying the critical parameters influencing enzyme production using the OFAT approach. The selected strain was found to produce a complete cellulolytic enzyme system (Fig. 2a). β-Glucosidase production using office paper waste as the substrate was promising, with maximum activity achieved after only three days of incubation. This was shorter than the maximum production times of FPase and CMCase, which peaked after four and six days of incubation, respectively.

Our results are consistent with a previous study indicating that β-glucosidase and additional cellulases (FPase and CMCase) were produced from *A. oryzae* and *A. niger* that were cultivated under solid-state fermentation (SSF) throughout time. On the tenth day, the highest amount of β-glucosidase was produced, and the activities of FPase and CMCase followed a similar trend, increasing in tandem with the incubation period. However, after 8 and 14 days, respectively, the highest levels of CMCase and FPase activity were observed [42]. Xin and Geng [43] showed the maximum amount of β-glucosidase produced by *T. reesei* after 8 h of growth on woodchips, and from *Colletotrichum graminicola* after 7 days of incubation [44]. However, Ng et al. [32] reported the maximum β-glucosidase activity from *Penicillium citrinum* YS40-5 when it was grown on rice bran for 4 days under SSF. On the other hand, the production of β-glucosidase from *Aspergillus niger* was noted to reach its maximum level after 12 days under submerged fermentation [45]. Additionally, under submerged fermentation (SmF) conditions (40 °C, pH 5.5, 120 rpm) with 10% (v/v) inoculum and 0.1% Tween-20, the *Aspergillus sydowii* isolate achieved maximum activity on the 6th day for all tested enzymes: filter paper activity (1.33 IU/ml), endoglucanase (1.32 IU/ml), exoglucanase (3.99 IU/ml), and β-glucosidase (9.24 IU/ml) [46]. On the other hand, submerged fermentation of the *Aspergillus niger* CDBB-H-175 strain yielded maximum extracellular β-glucosidase production at 31 days [47].

##### Effect of initial pH values on enzyme production

One of the most important environmental factors influencing mycelia growth, enzyme synthesis, and the movement of different components across the cell membrane is the pH of the medium [48]. β-glucosidase production by *Aspergillus niger* S1 was also tested at different pH values ranging from 5.0 to 7.0 and the optimum pH to produce β -glucosidase was found at pH 6.5 with the highest enzymatic activity (1.84U/ml) under liquid state fermentation that indicated in the Fig. 2b. These results are closer to β-glucosidase that was produced from *Aspergillus niger* CDBB-H-175 at pH 6 under submerged fermentation [47]. Under submerged fermentation, four distinct strains of the *Aspergillus* species produced extracellular β-glucosidase best at a pH of 6.0 [49], which is in close agreement with the optimum pH (5.5) reported for *Aspergillus sydowii* β-glucosidase production under submerged fermentation [46]. Additionally, Noor El-Deen et al. [42] stated the production of β-glucosidase from *A. niger* NRC 7 A and *A. oryzae* NRRL 447 at pH 6.0 under SSF, while 5.0 was the ideal pH for SSF to produce β-glucosidase, β-xylosidase, and xylanase by *Colletotrichum graminicola* [44]. On the other hand, applying submerged fermentation, *Trichoderma reesei* β-glucosidase reached its maximum production rate at pH 3.68 [50].

##### Effect of nitrogen sources on enzyme production

The impact of the various nitrogen sources on the production of β-glucosidase by *Aspergillus niger* S1 was examined and displayed in Fig. 2c. The best organic nitrogen source causing the maximum production of β-glucosidase from *Aspergillus niger* S1 was found to be yeast extract. The positive influence of yeast extract supplementation can be attributed to its role as a rich source of amino acids, peptides, vitamins, and trace elements that support fungal growth and secondary metabolite production. Consistent with our findings, previous studies also reported yeast extract as the optimal nitrogen source for maximum β-glucosidase production in *Colletotrichum graminicola* [44] and *Aspergillus niger* SOI017 [51]. However, the best nitrogen source for the production of β-glucosidase from *Chaetomium globosum* DX-THS3 was peptone [52]. Additionally, the production of β-glucosidase from *A. niger* NRC 7 A and *A. oryzae* NRRL 447 was increased by soybean flour as a nitrogen source [42]. Furthermore, Hamza and Sayadi [45] reported that a combination of wheat peptone and urea as the nitrogen source yielded the highest β-glucosidase production from *Aspergillus niger*. Moreover, Venturi et al. [53] found that the production of β-glucosidase from *Chaetomium thermophilum* var *coprophilum* was high when it was grown on peptone and yeast extract as a nitrogen source, whereas the highest level of production of extracellular β-glucosidase from *Penicillium citrinum* YS40-5 was obtained when cultured on urea as a nitrogen source under SSF [32]. During the optimization of submerged fermentation, a 0.5% concentration of yeast extract proved to be the optimal nitrogen source for producing acidophilic β-glucosidase from the mangrove soil isolate *T. reesei* [50].

##### Effect of incubation temperature on enzyme production

**Fig. 3:**
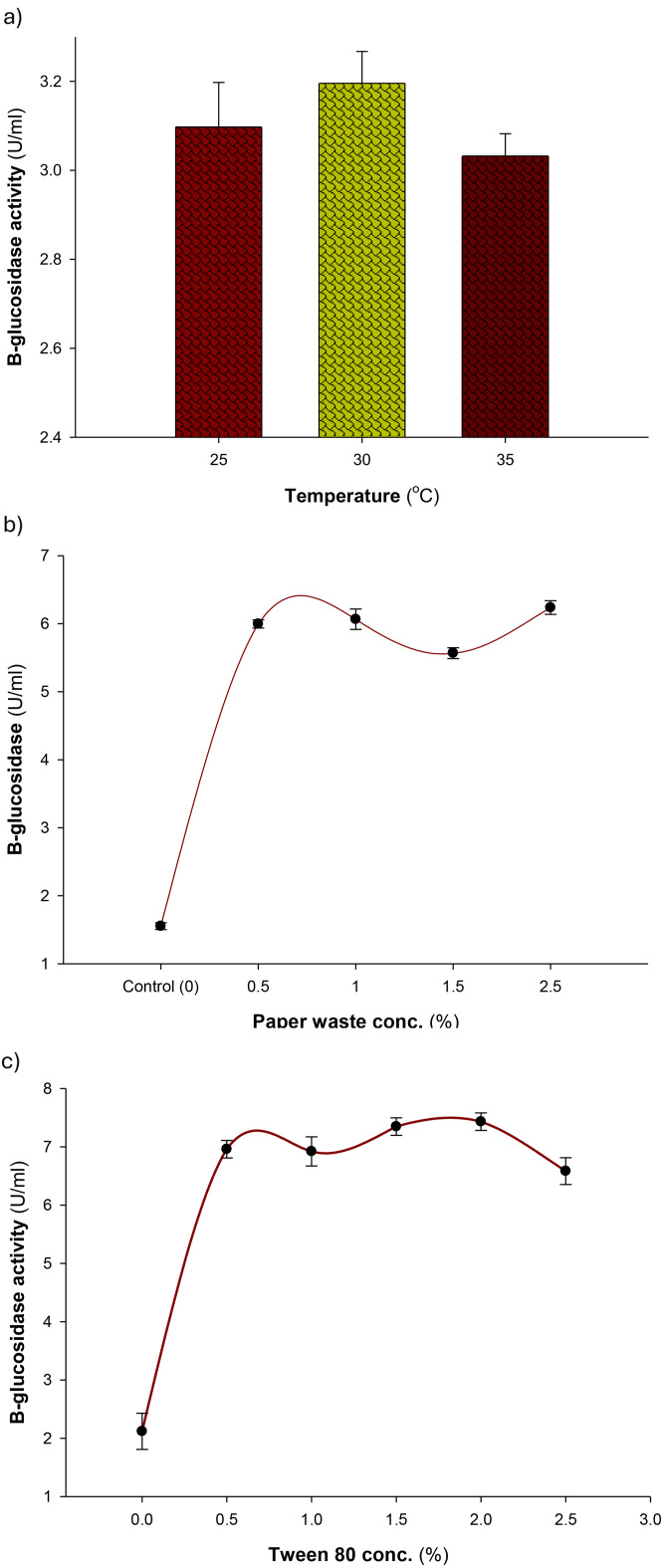
(**a**) Effect of temperature, (**b**) office paper waste concentration, and (**c**) Tween 80 concentration on the production of β-glucosidase by the newly isolated *Aspergillus niger* S1. Error bars represent mean ± standard deviation

The results obtained indicated that the optimum temperature to produce β–glucosidase from *Aspergillus niger* S1 is 30 °C (Fig. 3a). Enzyme activity is significantly influenced by temperature, where high temperatures can denaturize proteins, while low temperatures may affect the rate of enzyme catalysis [54]. Our findings are comparable to the ideal temperature (30 °C) for submerged fermentation, which produces β-glucosidase from four distinct *Aspergillus* strains [49]. Furthermore, the optimal temperature for the production of β-glucosidase from *Penicillium purpurogenum* KJS506 was at 32 °C [55]. Additionally, the production of β-glucosidase from non-Saccharomyces yeast strains was relatively higher, where it was found at 40 °C [54], which resembles to optimum temperature value (40 °C) recorded with *Aspergillus sydowii* β-glucosidase production under submerged fermentation [46]. In contrast, the Sun et al. [50] reported that 28 °C was the optimum incubation temperature for *T. reesei* β-glucosidase production under submerged fermentation conditions.

##### Effect of office paper waste concentration on enzyme production

The best concentration of office paper waste as a carbon source was found to be 1.5%, which resulted in the maximum production of β-glucosidase by *Aspergillus niger* S1 (Fig. 3b). The maximal activity of β-glucosidase produced from *Penicillium purpurogenum* KJS506 was obtained when rice straw was used as a carbon source [55]. Dhake and Patil [56] declared that growing *Penicillium Purpurogenum* on a medium with 1% sucrose as a carbon source produced the best amount of β-glucosidase [56]. Nevertheless, wheat bran (1%, w/w) was the most effective carbon source for *Colletotrichum graminicola*’s β-glucosidase production under SSF [44].

##### Effect of surfactant on enzyme production

The results obtained indicated that the best concentration of Tween 80 for β-glucosidase production by *Aspergillus niger* S1 was 1.5% (v/v) (Fig. 3c). This result is comparable to the synthesis of β-glucosidase in *Sporotrichum thermophile*, where an increase in Tween 80 concentration up to 1.5% (v/v) boosted the production of the enzyme. Because it affects the permeability of the cell membrane or interferes with the nonspecific binding of enzymes to substrates, Tween 80 in the medium enhances the synthesis of enzymes, which promotes enzyme desorption and recycling [57]. Additionally, Tween 80 enhanced the generation of β-glucosidase from the *Aspergillus terreus* strain EMOO 6 − 4 [58]. Additionally, optimum production of *Aspergillus sydowii* β-glucosidase under submerged fermentation was achieved at 0.1% Tween-20 [46].

#### Response surface methodology (RSM)

The outcomes of the CCD experimental tests are shown in Table 1 and were contrasted with the predicted values. To comprehend the data and their relationships, a quadratic model was employed. The following equation is expressed in terms of coded components and connects the response (β-glucosidase activity) to the CCD parameters (Incubation time (A), pH value (B), office paper waste (C), and yeast extract concentrations (D):

$$\begin{aligned} & \beta-Glucosidase activity\left( {{U \over {mL}}} \right) \cr& = 2.544 - 0.155A - 0.047B\cr& + 0.052C - 0.223D - 0.062AB\cr&+ 0.026AC - 0.068AD - 0.101BC\cr& + 0.201BD - 0.133CD - 0.205{A^2}\cr& + 0.060{B^2} - 0.088{C^2} - 0.027{D^2} \end{aligned}$$

**Fig. 4:**
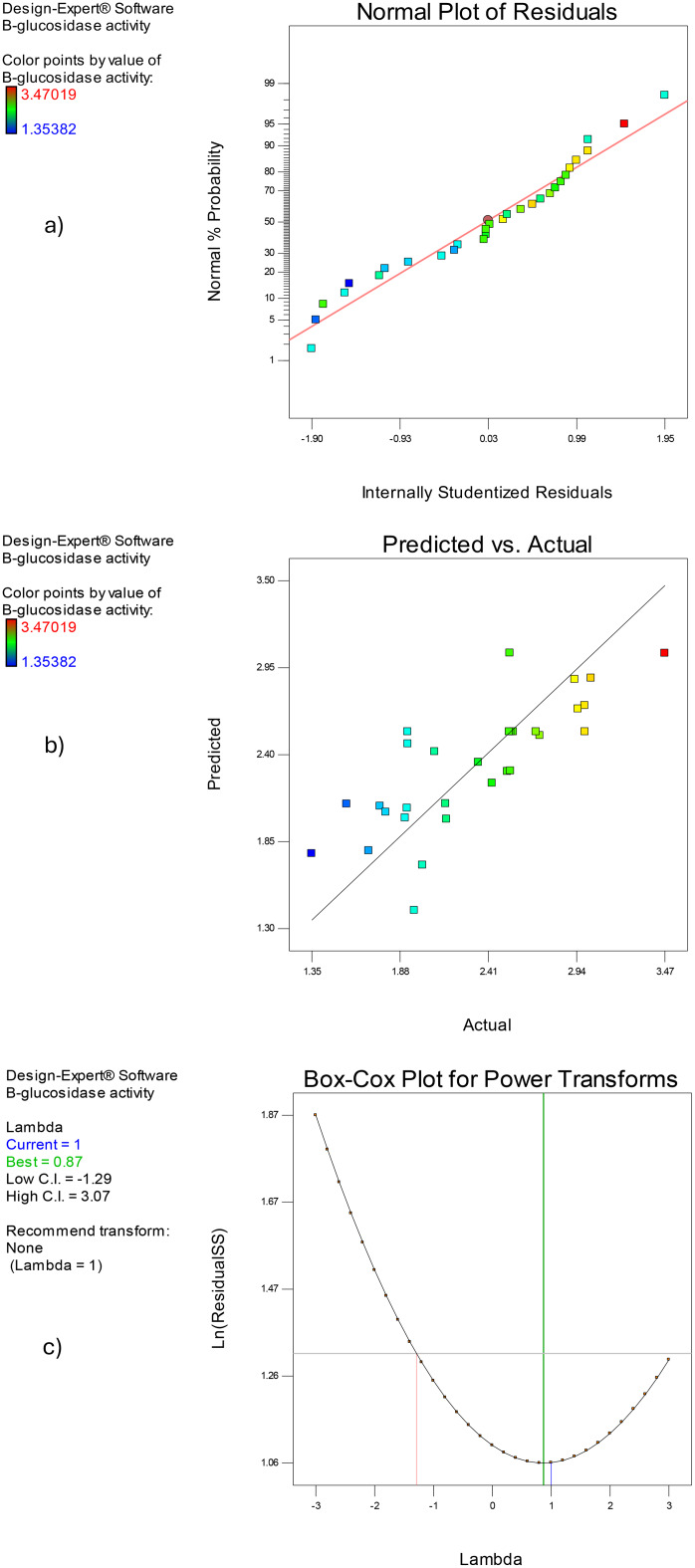
(**a**) The residuals’ normal plot. (**b**) The correlation between enzyme activity values predicted by the model and actual experimental values. (**c**) The current quadratic model’s Box-Cox power transformation diagram

Multiple regression analysis was used to examine the data (Table 1) and determine the expected β-glucosidase responses. Table 2 shows the results of the analyses of variance (ANOVA) for the response surface quadratic models. The models predicted enzymatic activity as a function of pH, incubation period, office paper waste, and yeast extract based on the F test. The models’ importance is further demonstrated by the low P-values, where D and A^2^ were shown to be significant model terms. The residual analysis, including comparisons with the incubation period, pH value, amounts of office paper waste, and yeast extract concentration, provides further detail on the developed RSM model. A key component of the RSM test is residual analysis, which verifies the model’s assumptions. Since the points form a linear graph, the residuals’ normal plot (Fig. 4a) shows that the residuals are normally distributed. In our analysis, the residuals are confirmed to follow a normal distribution, as indicated by the straight-line pattern in the plot of residuals versus internally studentized residuals. This demonstrates that the anticipated values from the enzyme production model and the actual experimental findings (Fig. 4b) coincide rather well. The residuals vs. run number figure, which showed points dispersed about zero, demonstrated that the model fits the data. So, the predicted vs. actual plot revealed that the predicted and experimental results were in good correlation (Fig. 4b). For the model to be validated, the correlation between anticipated values and actual experimental outcomes is essential [5]. Using the Box-Cox transformation, selecting a suitable exponent (Lambda = λ) improves the residual normality of the quadratic model [59]. Additionally, a Box-Cox plot is a useful tool for figuring out the best power transformation [5, 37]. The Box-Cox power transformation plot for enzyme production optimization using the current quadratic model is displayed in Fig. 4c. The current study proved that there was no need for any additional changes because the current lambda (= 1) was adequate (Fig. 4c).

**Table 2:** Analysis of variance (ANOVA) for Response Surface Quadratic Model of ß-glucosidase activity

Source	Sum of Squares	df	Mean Square	F Value	*p*-value Prob > F
Model	4.637	14	0.331	1.721	0.154
A-Incubation time	0.579	1	0.579	3.007	0.103
B-pH value	0.052	1	0.052	0.272	0.609
C-Office paper waste	0.064	1	0.064	0.334	0.572
D-Yeast extract	1.193	1	1.193	6.196	0.025
AB	0.062	1	0.062	0.324	0.577
AC	0.011	1	0.011	0.055	0.817
AD	0.073	1	0.073	0.381	0.546
BC	0.163	1	0.163	0.845	0.372
BD	0.649	1	0.649	3.370	0.086
CD	0.284	1	0.284	1.476	0.243
A^2^	1.149	1	1.149	5.968	0.027
B^2^	0.097	1	0.097	0.505	0.488
C^2^	0.211	1	0.211	1.097	0.312
D^2^	0.020	1	0.020	0.101	0.755
Residual	2.887	15	0.192		
Lack of Fit	2.285	10	0.229	1.897	0.249
Pure Error	0.602	5	0.120		
Cor Total	7.524	29			

R^2^ 0.6163, Adj R^2^ 0.2582^,^ Std. Dev. 0.439, Adeq Precision 5.245, Mean 2.34, C.V% 18.78, PRESS 14.03

Multiple regression analysis was used to examine the CCD resulting response (β-glucosidase activity) data. The analysis of variance (ANOVA) for the response surface quadratic models were reported as a function of pH, incubation period, office paper waste, and yeast extract based on the F test

**Fig. 5:**
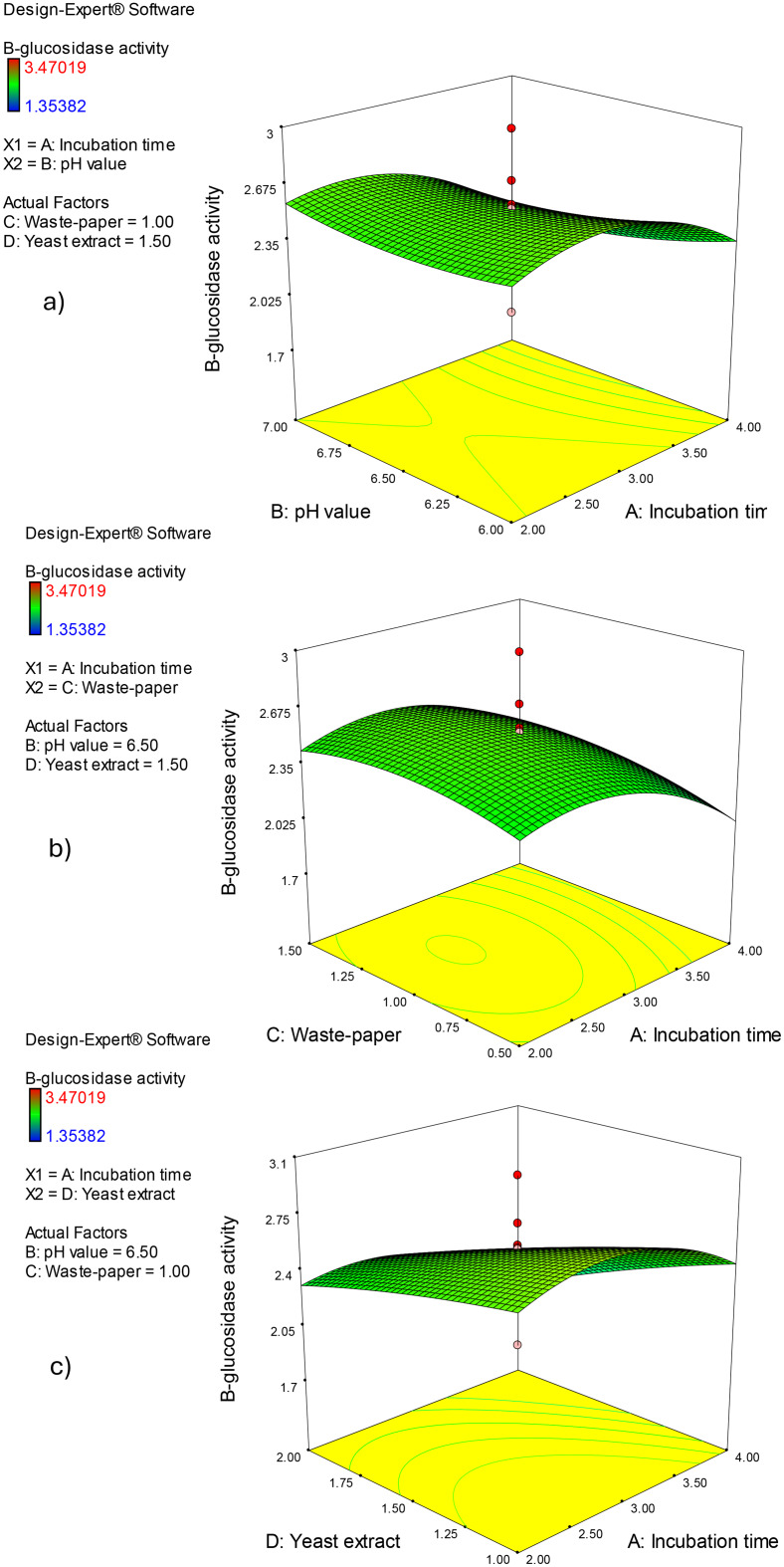
3D surface plots of ß-glucosidase activity using the CCD method. The two active parameters have an impact on the findings that are shown, whereas the other parameters are kept at center values (Incubation period (**A**), 3 days; pH value (**B**), pH 6.5; office paper waste concentration (**C**), 1%; and yeast extract concentration (**D**), 1.5%)

**Fig. 6:**
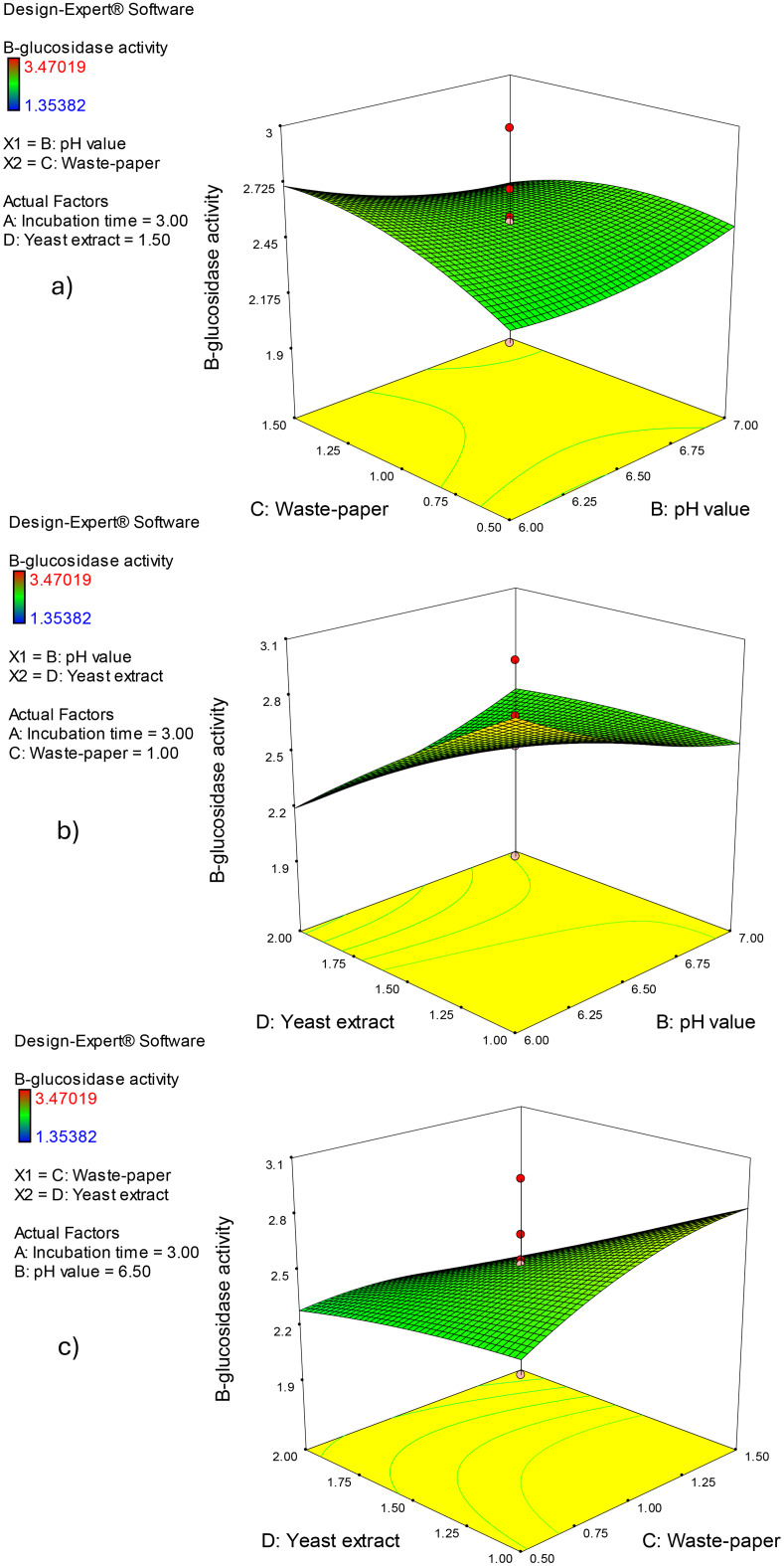
3D surface plots of ß-glucosidase activity using the CCD method. The two active parameters have an impact on the findings that are shown, whereas the other parameters are kept at center values (Incubation period (**A**), 3 days; pH value (**B**), pH 6.5; office paper waste concentration (**C**), 1%; and yeast extract concentration (**D**), 1.5%)

**Fig. 7:**
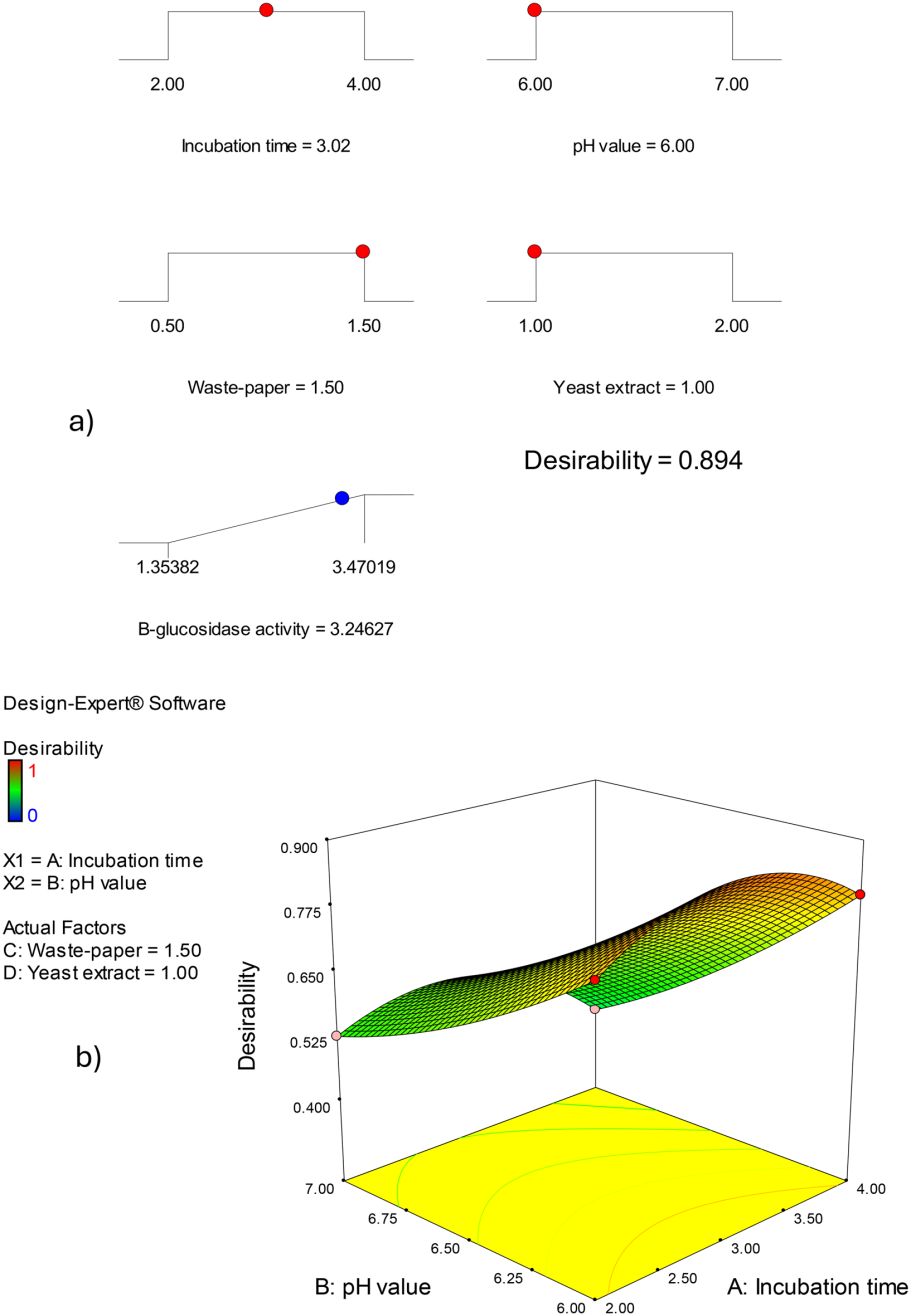
The desirability ramps (a) and 3D plot (b) for the β-glucosidase enzyme production optimization. The combination of 3 days of incubation period (**A**), pH value of 6.0 (**B**), 1.5% (w/v) office paper waste (**C**), and 1 g of yeast extract (**D**) was recommended

Three-dimensional surface plots (Figs. 5 and 6) illustrate the influence of the assessed parameters on β-glucosidase enzyme production, providing a holistic view of the data’s patterns and relationships. The graphs were displayed for two of the four parameters at different interconnect values, while the other two parameters stayed constant midway (Incubation period (A), 3 days; pH value (B), 6.5; office paper waste concentration (C), 1%, and yeast extract concentration (D), 1.5%). β-glucosidase enzyme production at constant concentration of office paper waste (C; 1%), and yeast extract (D; 1.5 g/L) was investigated in Fig. 5a concerning incubation period (A) and pH value (B). The β-glucosidase enzyme production has increased dramatically with the incubation period in days (A) up to 3 days, then decreased. The effect of different incubation periods (A) and office paper waste concentrations (C) on β-glucosidase enzyme production by *Aspergillus niger* S1 at predetermined values of pH (B, pH 6.5) and office paper waste concentration (C, 1%) was displayed in Fig. 5b. As demonstrated by the resultant response design, increasing the incubation time causes the ß-glucosidase production to rise concurrently for up to 3 days. Figure 5c shows the effects of the interaction between the incubation period (A) and yeast extract concentration (D) at constant concentrations of pH value (B, pH 6.5) and office paper waste concentration (C, 1%). The resulting response curve shows that ß-glucosidase synthesis was slightly repressed by the increase in yeast extract concentration, whereas the impact of incubation time was still maximized at 3 days (Fig. 5c). The impact of the concentrations of pH value (B) and office paper waste concentration (C) at fixed incubation period (A), 3 days; and yeast extract concentration (D), 1.5% were depicted in Fig. 6a. Plotting the direct effects of both factors on ß-glucosidase production by *Aspergillus niger* S1 shows that it rises as both parameters’ concentrations increase up to 1.5% office paper waste and pH 7.0 (Fig. 6a). Furthermore, Fig. 6b highlights the negative effect of increasing the pH value (B) and yeast extract concentration (D). Finally, Fig. 6c illustrates a little increase over the midpoints of office paper waste concentration (C, 1%) resulted in an elevation in *Aspergillus niger* S1 ß-glucosidase synthesis (Fig. 6c). Optimization ramps based on CCD technique findings indicate a β-glucosidase enzyme production of 3.25 U/mL for a production combination of 3 days of incubation period (A), pH value of 6.0 (B), 1.5% (w/v) office paper waste (C), and 1 g of yeast extract (D) (Fig. 7a, b). Three independent biological replicates were carried out, which was statistically indistinguishable from the model-predicted value (*p* > 0.05, one-sample t-test). To validate the theoretical optimal point predicted by the quadratic model (3.25 U/mL), a confirmatory experiment was conducted using the recommended optimal conditions (3 days of incubation period, pH value of 6.0, 1.5% (w/v) office paper waste, and 1 g of yeast extract). The experimental result obtained was in close agreement with the predicted value (higher by ~ 5%), confirming the model’s robustness and accuracy.

### Bio-deinking for paper making using *Aspergillus niger* S1 β-glucosidase

**Fig. 8:**
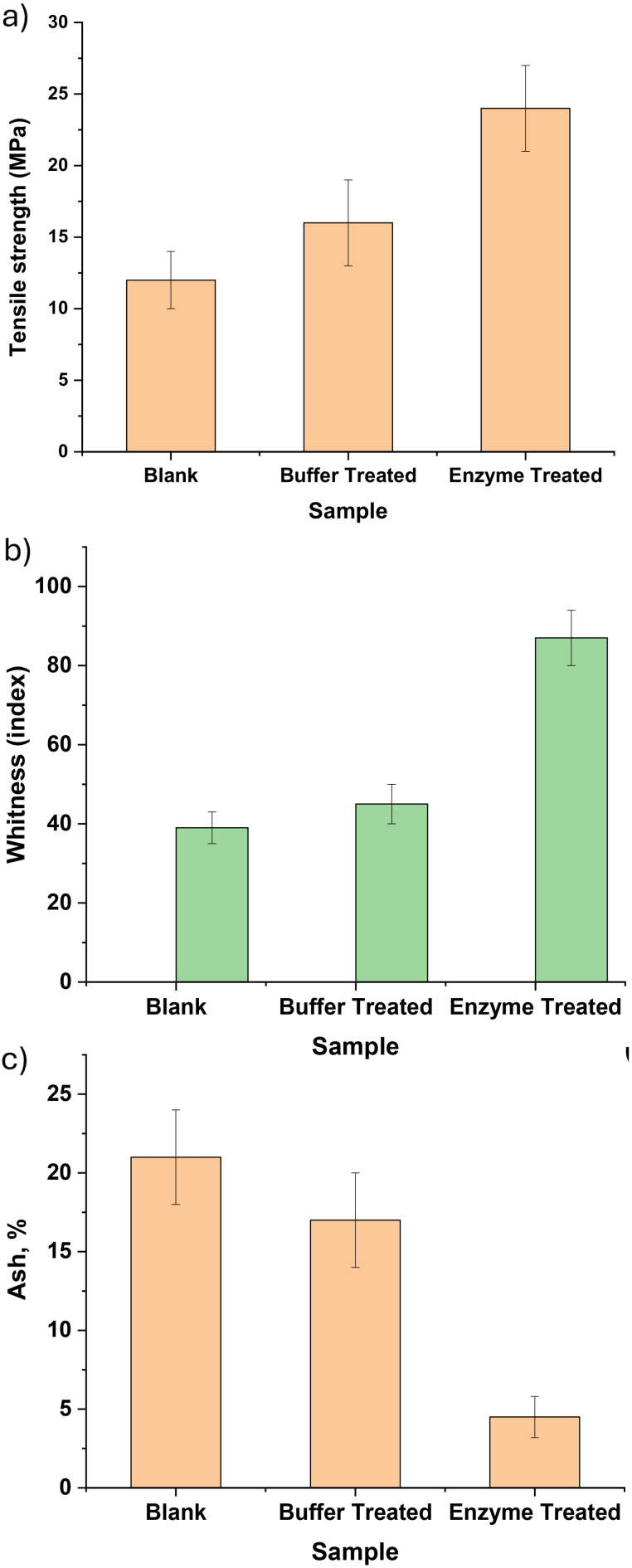
(**a**) Tensile strength of the blank OPW and treated OPW with buffer and crude enzyme. (**b**) Whiteness index of the blank OPW, treated with buffer and crude enzyme. (**c**) Ash content of blank OPW and treated OPW using buffer and crude enzyme (*n* = 3 replicates), where error bars represent mean ± standard deviation

The ability of the β-glucosidase crude enzyme to deink the MOWP was evaluated using the whiteness index, tensile strength (MPa), and ash content (%). Tensile strength is one of the judged aspects of the ability of the crude enzyme to eliminate the ink particles and increase the attraction between the fibers, which increases the mechanical properties [60]. The untreated OPW (blank) sample observed low tensile strength around 12 ± 2 MPa, as presented in Fig. 8a. However, the buffer OPW sample observed an improvement to about 16 ± 3 MPa, and the enzyme-treated sample was increased by twofold to about 24 ± 3 MPa. These observations affirmed that the ink particle interrupted the cellulose fibers attraction, and the buffer acted as a washing effect that slightly improved the mechanical properties. Furthermore, the enzyme treatment was illustrated as highly efficient in the tensile strength improvement that could be reflected in the attraction of the paper sheets fibers in the absence of the ink particles (Fig. 8a).

Figure 8b shows the witness index of the treatment of the OPW using buffer as a positive control and crude β-glucosidase compared with blank OPW. The effect of the crude enzyme on the whiteness index was noted as a high index (about 87 ± 7). On the other side, the blank sample recorded 39 ± 4, and the sample treated with buffer recorded 45 ± 5, with no significant improvement in whiteness index. Whereas the crude enzyme affected the cellulose fiber with the elimination of the fine end cellulosic fibers that were attached with ink particles, and deinking ability was recorded as 123% for the enzyme-treated sample, as well as 15.4% for the treated sample [25]. Figure 8c shows the ash content of prepared paper sheets. Hence, the ash content of the analyzed samples is due to the pulp itself, as well as the inorganic part of the ink. The blank OPW showed a high ash content of around 21 ± 3%, which decreased to about 17 ± 3% after treatment with buffer. The crude enzyme acted as a stronger element to the inorganic part of the paper sheet, where the ash content was decreased to about 4.5 ± 1.3% 60 [61].

Moreover, the digital photos of the making of paper sheets with different treatment processes compared to the blank ones were illustrated in Supplementary Fig. S1. The paper sheets presented ink particles in the blank paper sheet that disappeared after treatment with the enzyme. The treatment sample with buffer presented moderate ink particle distribution. Otherwise, the enzyme-treated samples presented a clean appearance. The conclusion of the biodeinking analysis emphasized that the buffer could act as a washing process, whereas the enzyme treatment delicately deinks via β-glucosidase activity (Fig. S1).

## Conclusion

This study conclusively demonstrates the successful development and optimization of a sustainable process for β-glucosidase production using the newly isolated fungal strain *Aspergillus niger* S1. By employing office paper waste as a low-cost carbon source, the research establishes an effective model for the valorization of lignocellulosic waste. Systematic optimization, culminating in a central composite design, identified precise cultivation parameters (1.5% OPW, 1 g/L yeast extract, pH 6.0, 3-day incubation) to achieve a maximum enzyme activity of 3.25 U/mL. Crucially, the practical application of the produced β-glucosidase in a biodeinking process confirmed its significant technical efficacy. The enzymatic treatment not only facilitated the effective removal of ink particles but also led to substantial improvements in the quality of the recycled paper, notably enhancing tensile strength and brightness while reducing ash content. In summary, this work presents an integrated, environmentally benign biotechnology that aligns with circular economy principles. It transforms paper waste into a valuable biocatalyst, which in turn enhances the recycling process itself. The findings highlight a promising and sustainable alternative to conventional chemical deinking, addressing key industrial demands for greener waste management and resource-efficient practices in the paper recycling sector.

## Supplementary Information

Below is the link to the electronic supplementary material.

Supplementary Material 1

## Data Availability

On reasonable request, the corresponding author can supply the datasets used and/or analyzed in this study. With the accession number PV230693, the identified *Aspergillus niger* isolate S1 was registered in the GenBank (NCBI) database.
