# Supplementary figures and images for: Enhanced production and biodeinking application of β-glucosidase from Aspergillus niger S1 via submerged fermentation using office paper waste through conventional and statistical approaches
Source: BMC Biotechnol. 2026 Apr 13;26:46. doi: 10.1186/s12896-026-01139-9 (PMC13078100; doi:10.1186/s12896-026-01139-9)

| 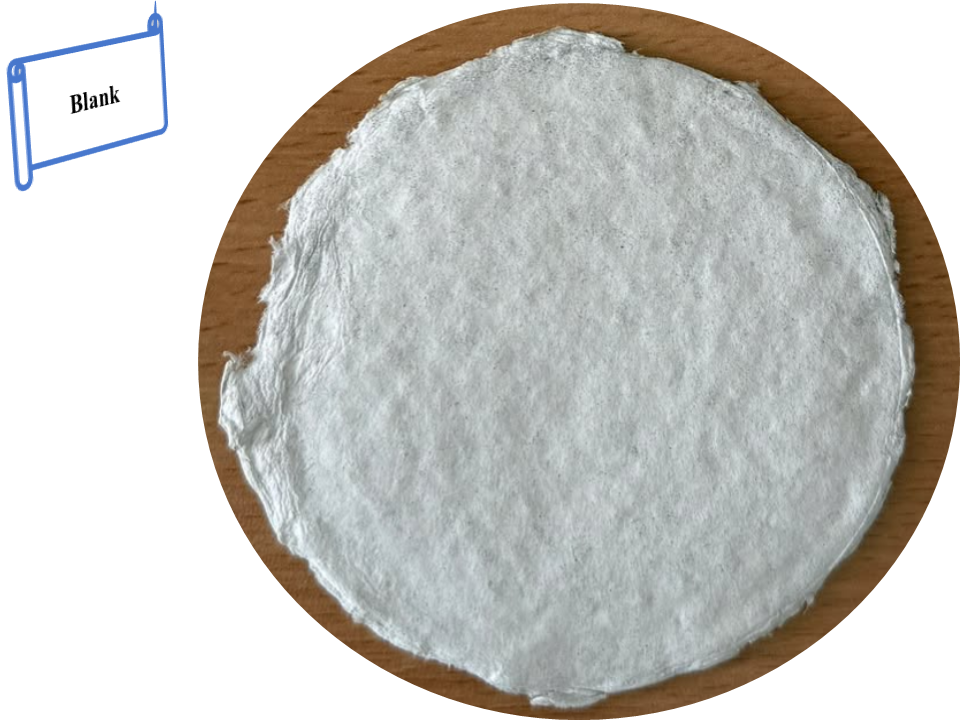 |
| --- |
| 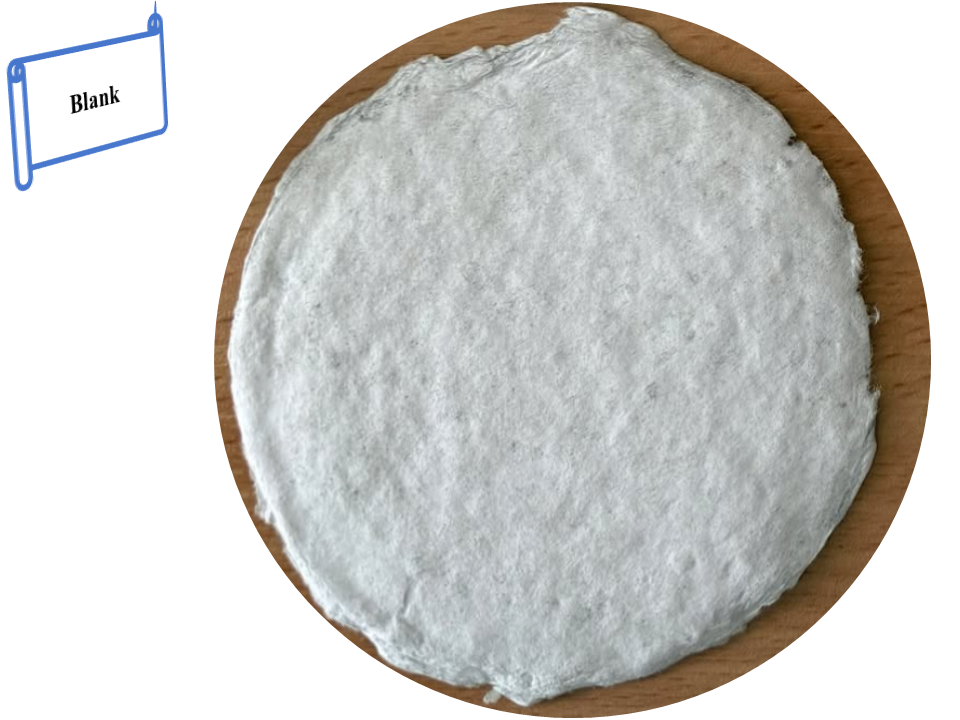 |
| 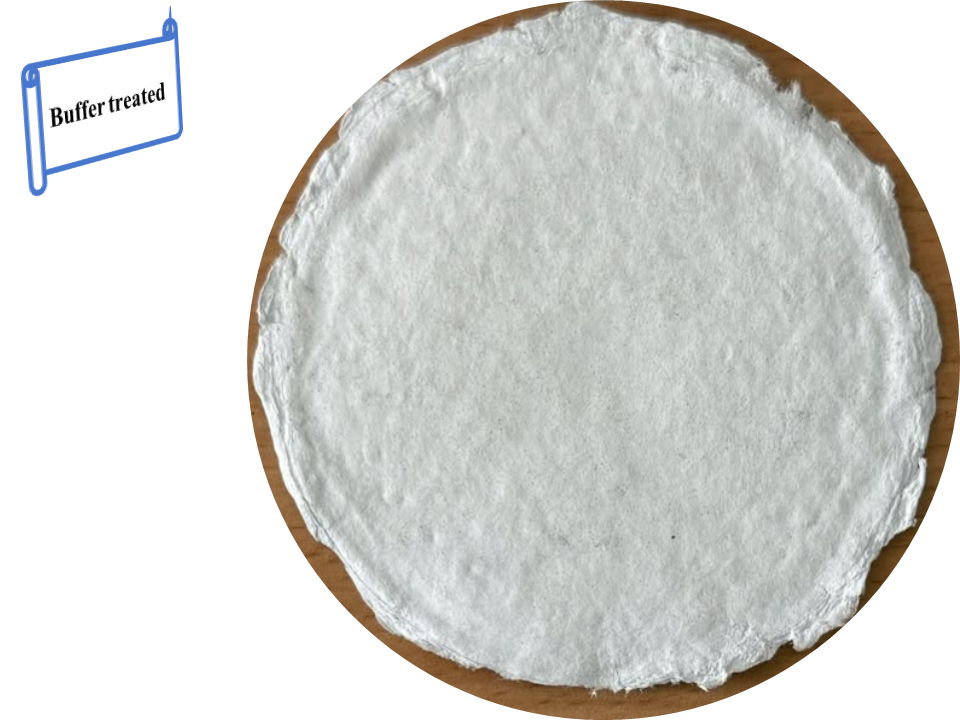 |
| 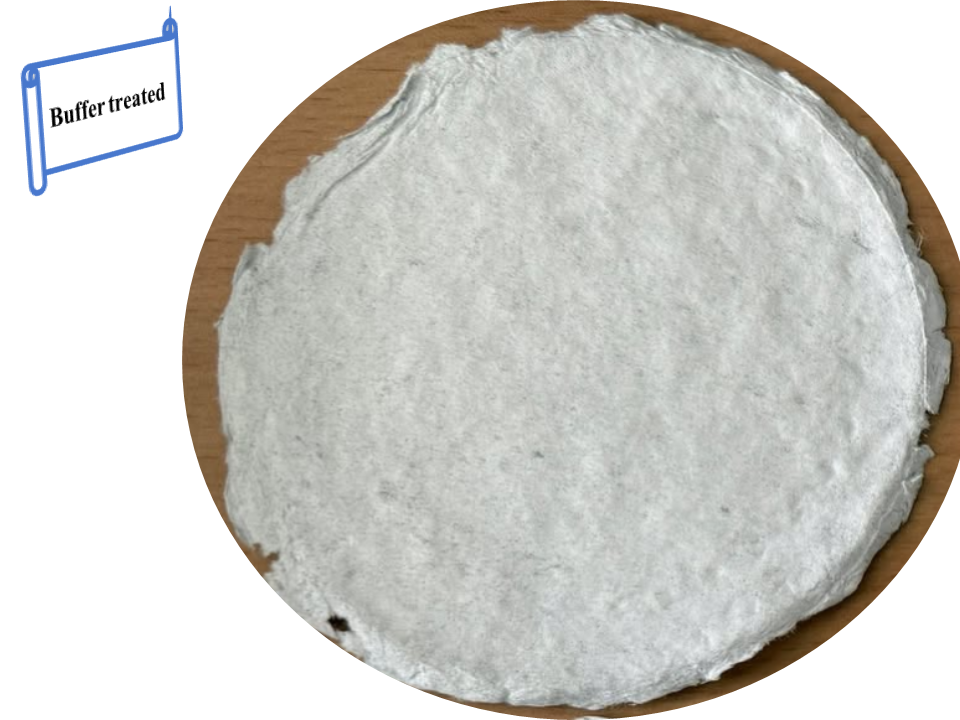 |
| 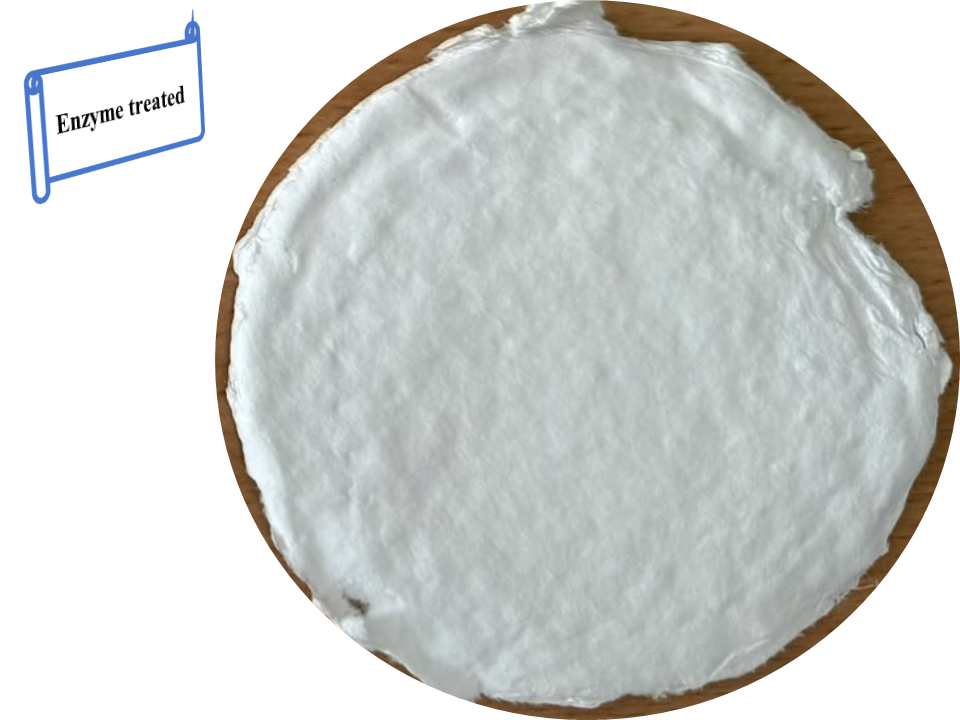 |
| 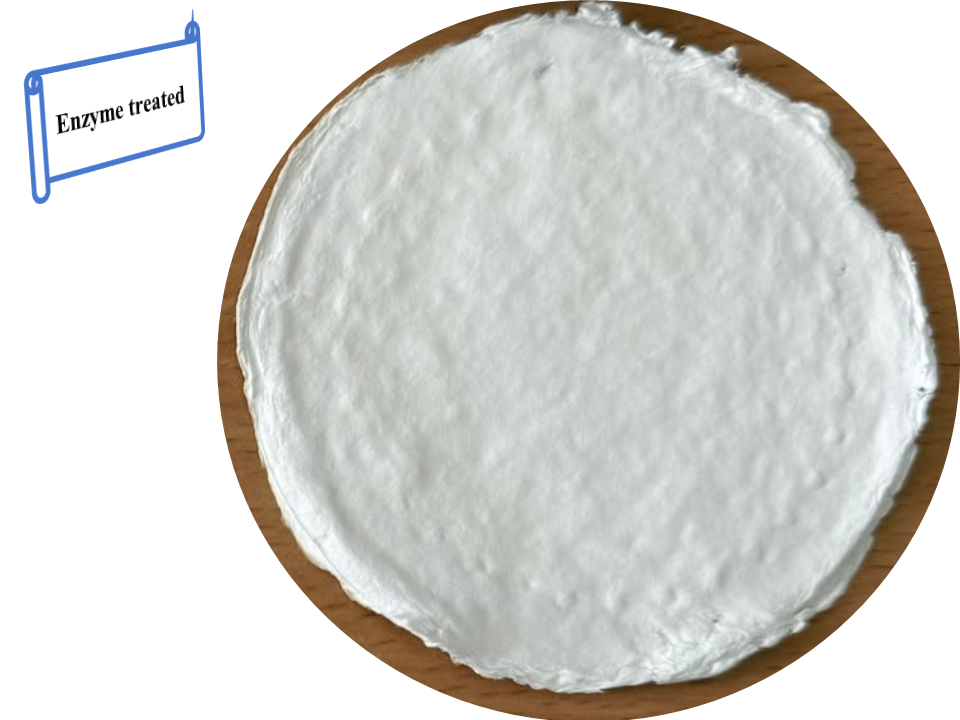 |
| **Fig. (S1):** Digital photos of the blank, buffer, and enzyme-treated paper sheets. |

Supplement: Supplementary file 1 — Supplementary Material 1 [file 12896_2026_1139_MOESM1_ESM.docx]
